# Evolution of Well-Being and Associated Factors during the COVID-19 Pandemic

**DOI:** 10.62641/aep.v53i1.1783

**Published:** 2025-01-05

**Authors:** Encarnación Martínez-Mondéjar, María Falcon-Romero, Carmen Rodríguez-Blazquez, María Romay-Barja, María João Forjaz, Lucía Fernández-López, Olga Monteagudo-Piqueras

**Affiliations:** ^1^Medicina Preventiva y Salud Pública, Servicio Murciano de Salud, 30008 Murcia, Spain; ^2^Medicina Legal, Departamento de Ciencias Sociosanitarias, Universidad de Murcia, 30120 Murcia, Spain; ^3^Centro Nacional de Epidemología, Instituto de Salud Carlos III, 28029 Madrid, Spain; ^4^Centro Nacional de Medicina Tropical, Instituto de Salud Carlos III, 28029 Madrid, Spain; ^5^Departamento de Farmacología, Universidad de Murcia, 30120 Murcia, Spain; ^6^Servicio de Promoción y Educación para la Salud, Dirección General de Salud Pública y Adicciones, Consejería de Salud de la Región de Murcia, 30008 Murcia, Spain

**Keywords:** COVID-19, risk of depression, social determinants of health, vulnerable populations, well-being

## Abstract

**Background:**

The COVID-19 pandemic was a global public health crisis with an unparalleled impact worldwide, presenting a significant challenge for both physical and mental health. The main objective of this study was to analyze the risk of depression during the COVID-19 pandemic and how this was affected by sociodemographic factors, pandemic fatigue, risk perception, trust in institutions, and perceived self-efficacy.

**Methods:**

A cross-sectional study was conducted in the Region of Murcia through two online surveys completed by 1000 people in June 2021 (Round 1) and March 2022 (Round 2). Risk of depression was measured using the 5-item World Health Organization Well-Being Index (WHO-5) questionnaire, and descriptive, bivariate, and multivariate regression analyses were performed to identify factors associated with the risk of depression.

**Results:**

In Round 1, 35.2% of the sample presented a risk of depression, which increased to 39.1% in Round 2. Those at greater risk were women, individuals with lower socioeconomic status, those with less family support, lower trust in institutions, higher perceived risk of contracting the disease, and higher levels of pandemic fatigue.

**Conclusions:**

Identifying vulnerable populations facing mental health issues can help the authorities and institutions that are responsible for managing public health crises to develop and implement inclusive strategies and interventions tailored to the population's needs.

## Introduction

The COVID-19 pandemic was a global public health emergency with unprecedented worldwide impact, posing a significant challenge to physical and mental health [1]. The deterioration of emotional well-being occurred globally, with Spain being one of the countries with high prevalence rates of anxiety, stress, depression, and/or insomnia during the COVID-19 pandemic [2, 3, 4, 5]. Moreover, the World Health Organization (WHO) states that 93% of countries experienced disruptions or impacts on their mental health services due to the pandemic [6].

This decline in mental health could be attributed to numerous factors: the scale and speed at which the pandemic developed, the fear of contagion for oneself and loved ones, job loss or financial concerns, lack of trust in institutions, restrictive control measures adopted by authorities, lack of information or alarming information, and pandemic fatigue, among others [7, 8, 9, 10, 11].

In this context, it is important to identify the varying degrees of vulnerability to the psychological impact caused by the pandemic across different population groups, especially in relation to gender, age, and socioeconomic level. Behavioral and cultural insights studies are crucial to understanding drivers and barriers of well-being in times of crisis [12] and can help to identify the population’s needs and to design health strategies aimed at reducing inequalities generated in emergencies such as the COVID-19 pandemic [13].

The aim of this study was to measure the subjective well-being of the general population and evaluate the influence of sociodemographic factors, social support, pandemic fatigue, risk perception, trust in institutions, and perceived self-efficacy.

## Data and Methods

### Procedure

The study protocol was based on the WHO behavioral insights tool included in the COVID-19 Snapshot Monitoring (COSMO) initiative [14].

This was a cross-sectional study conducted in the Region of Murcia through two separate online surveys, the first in June 2021 (Round 1) and the second in March 2022 (Round 2), coinciding with the end of the two periods of highest COVID-19 incidence in the Region of Murcia [15]. The surveys were conducted with two independent samples of 1000 people deemed to be representative of the population of the region in terms of age, gender, and area of residence. The inclusion criteria required respondents to be 18 years or older and living in the Region of Murcia. The exclusion criteria consisted of not meeting the inclusion criteria or not signing the informed consent form. The surveys were carried out by a social research company, using email invitations sent to panel members who met the selection criteria. Non-respondents were replaced by others from the same stratum. This study was approved by the Ethics Committee of Hospital Clínico Universitario Arrixaca (CEI 2020-11-23-HCUVA).

### Measurements

The variables used were adapted from the guide proposed by the WHO to analyze the factors influencing preventive behavior [14] and its national adaptation through the COSMO-SPAIN project [16]. They included sex (male/female), age, education level, area of residence (urban >20,000 inhabitants/rural ≤20,000 inhabitants), perceived socioeconomic level (low/middle/high), financial or work difficulties if stopped working as a consequence of the pandemic (yes/no), and support from family or friends for assistance during lockdowns or COVID-19 isolations (yes/no). Subjective well-being was assessed using the 5-item World Health Organization Well-Being Index (WHO-5) scale [17] which measures mood using five statements: I feel cheerful and in good spirits, I feel calm and relaxed, I feel active and vigorous, I wake up feeling fresh and rested, My daily life is filled with things that interest me. Each item has a scale of 0 (At no time) to 5 (All of the time). The WHO-5 is a recognized and validated screening tool to detect the risk of depressive disorder due to its high sensitivity and low specificity. People with a score ≤50 are classified as suspected of depression or at risk of depression, and a positive result indicates the need for a structured clinical evaluation to confirm a possible case [18, 19].

The degree of pandemic fatigue was measured using the COVID-19 Pandemic Fatigue Scale (CPFS), a seven-item scale validated in Spain. The score ranges from 5 to 30, with a higher score indicating a greater degree of pandemic fatigue [20]. Perceived self-efficacy was measured with the question, “For me, avoiding coronavirus/COVID-19 infection in the current situation is…”, with a response scale of 1 (very difficult) to 5 (very easy) [16]. The variable was dichotomized into low perceived self-efficacy (scores 1–3) and high perceived self-efficacy (scores 4–5). Risk perception was measured with the following questions: “What do you think are your chances of contracting coronavirus/COVID-19?” from 1 (very unlikely) to 5 (very likely); and “How severe do you think the illness would be for you if you contracted coronavirus?” from 1 (not severe at all) to 5 (severe) [16]. Both were dichotomized into low/high perceived risk of contagion/severity. The population’s level of trust in institutions was evaluated by asking, “How much trust do you have in the following institutions/organizations in addressing the challenges posed by COVID-19?” The institutions evaluated were the central government, the autonomous community, the workplace, the health center/hospital, and scientists. Scores were rated on a scale of 1 (no trust) to 5 (a lot of trust) [16]. The trust level was calculated by summing the raw values of the responses for each item and subsequently dichotomizing it using the median value into two categories: low and high trust.

### Statistical Analysis

Analysis was performed using IBM SPSS Statistics version 22 (IBM, Armonk, NY, USA). A descriptive statistical analysis of each sample was performed. Frequencies and confidence intervals (CIs) were calculated for categorical variables, and means and standard deviations (SDs) for continuous variables. The internal consistency of the scales was examined by calculating Cronbach’s alpha coefficient. Bivariate analysis was conducted using the chi-square test to detect statistically significant differences between categories, based on the suspicion of depression (WHO-5 ≤50 points). The statistically significant variables were used to perform multivariate logistic regression (considering *p* values of less than 0.05 to be statistically significant).

Model 1 was adjusted for the variables of gender, age group, and socioeconomic level. Model 2 was additionally adjusted for the variables of having family/social support and facing financial or work difficulties if they had to stop working due to the pandemic, as these are important contextual factors. Model 3 for Round 1 was also adjusted for the variables of pandemic fatigue, trust in institutions, and perceived risk of disease severity and probability. In Model 3 for Round 2, the variable of financial or work difficulties if having to stop working was removed as it was not statistically significant. In Model 4 for Round 2, the perceived risk of severity variable was also removed to adjust the model only for statistically significant variables.

## Results

### Descriptive Analysis of the Sample

The characteristics of the samples are detailed in Table 1. In Round 1, the mean score of the WHO-5 was 57.49 (SD: 21.9), and in Round 2 it was 55.18 (SD: 20.7). The Cronbach’s alpha was 0.92 and 0.903 for Round 1 and 2 respectively. Using the scale as a screening tool for suspected depression with a cutoff point of 50, 35.2% of the sample was at risk of depression in Round 1 and 39.1% in Round 2 (Table 1).

**Table 1.** **Characteristics of the sample in Round 1 (June 2021) and Round 2 (March 2022) and descriptive analysis of the variables**.

Characteristics		Round 1	Round 2
		(n = 1004)	(n = 1000)
		n (%)	n (%)
Sex	Male	490 (48.8)	487 (48.7)
	Female	514 (51.2)	513 (51.3)
Age (years)	18–34	247 (24.6)	327 (32.7)
	35–54	403 (40.1)	337 (33.7)
	≥55	354 (35.3)	336 (33.6)
Socioeconomic level	Low	484 (48.2)	204 (20.4)
	Medium	255 (25.4)	503 (50.3)
	High	265 (26.4)	293 (29.3)
Area of residence	Urban (>20,000 inhabitants)	825 (82.2)	553 (55.3)
	Rural (≤20,000 inhabitants)	179 (17.8)	447 (44.7)
Higher education level	No	566 (56.4)	392 (39.2)
	Yes	438 (43.6)	608 (60.8)
Family/friends support for assistance during lockdown	No	133 (13.2)	99 (9.9)
	Yes	871 (86.8)	901 (90.1)
Financial/work difficulties if stopped working	No	578 (57.6)	570 (57.0)
	Yes	426 (42.4)	430 (43.0)
Risk of depression screening	Yes (WHO-5 ≤50)	353 (35.2)	391 (39.1)
	No (WHO-5 >50)	651 (64.8)	609 (60.9)

***Note***: WHO-5, 5-item World Health Organization Well-Being Index.

### Bivariate Analysis

Table 2 shows the characteristics of those at risk of depression. When disaggregated by sex, in Round 1, 28.6% of men had suspected depression compared to 41.4% of women. In Round 2, the percentage of men with suspected depression rose to 32.2% and to 45.6% for women. According to the other variables analyzed, the population sample with the highest suspicion of depression in both rounds were those with a low socioeconomic level, those without support from family or friends during COVID-19 illness, those with a higher perception of the risk of the disease, and those with lower trust in institutions (Table 2).

**Table 2.** **Sociodemographic characteristics and perceptions by Round in people with suspected depression. Bivariate analysis**.

		WHO-5 ≤50 (suspected depression)			
		Round 1. n (%)	*p* value	Round 2. n (%)	*p* value
Sex	Male	140 (28.6)	<0.001	157 (32.2)	<0.001
	Female	213 (41.4)		234 (45.6)	
Age (years)	18–34	89 (36)		149 (45.6)	0.005
	35–54	160 (39.7)	0.012	130 (38.6)	
	≥55	104 (29.4)		112 (33.3)	
Socioeconomic level	Low	205 (42.4)	<0.001	113 (55.4)	<0.001
	Medium	78 (30.6)		211 (41.9)	
	High	70 (26.4)		67 (22.9)	
Higher education	No	209 (36.9)	0.183	161 (41.1)	0.305
	Yes	144 (32.9)		230 (37.8)	
Area of residence (inhabitants)	Rural (≤20,000)	54 (30.2)	0.123	165 (36.9)	0.203
	Urban (>20,000)	299 (36.2)		226 (40.9)	
Family/friends support for assistance during lockdown	No	69 (51.9)	<0.001	60 (60.6)	<0.001
	Yes	284 (32.6)		331 (36.7)	
Financial/work difficulties if stopped working	No	167 (28.9)		193 (33.9)	
	Yes	186 (43.7)	<0.001	198 (46)	<0.001
Risk perception severity	Low	83 (27.2)	<0.001	135 (34.3)	0.012
	High	270 (38.6)		256 (42.2)	
Risk perception probability	Low	138 (28.5)	<0.001	236 (35.2)	<0.001
	High	215 (41.4)		155 (47)	
Perceived self-efficacy	Low	163 (36.5)	0.438	250 (40.7)	0.186
	High	190 (34.1)		141 (36.5)	
Trust in institutions	Low	216 (61.2)	<0.001	230 (45.4)	<0.001
	High	137 (38.8)		161 (41.2)	

Note: WHO-5, 5-item World Health Organization Well-Being Index; *p* value for the chi-square test.

### Multivariate Logistic Regression for Risk of Depression

When applying multivariate analysis in the final model (Model 3) in Round 1, the results, which are detailed in Table 3, indicated that the risk of depression was higher in women than in men (odds ratio (OR) = 1.843; *p* value (*p*) < 0.001) and in people with a low socioeconomic level (OR = 1.758; *p* = 0.002) compared to those with a higher level. Having family support was a protective factor against the risk of depression (OR = 0.531; *p* = 0.002). Low trust in institutions indicated a higher risk of depression (OR = 1.523; *p* = 0.004) compared to high trust. A higher perception of risk, both of contagion and severity of COVID-19, indicated a higher risk of depression. Financial or work difficulties if stopped working indicated a higher risk of depression (OR = 1.478; *p* = 0.008) compared to not experiencing difficulties. With regard to pandemic fatigue, the odds ratio values are very close to the value of 1 (OR = 0.971; *p* = 0.018) so we cannot confirm an association between the variables.

**Table 3.** **Multivariate logistic regression of the risk of depression in Round 1**.

Risk of depression according to the WHO-5 in Round 1																			
		Model 1						Model 2						Model 3					
		B	SE	Wald	*p* value	OR	95% CI	B	SE	Wald	*p* value	OR	95% CI	B	SE	Wald	*p* value	OR	95% CI
Sex	Male	Ref.						Ref.						Ref.					
	Female	0.556	0.137	16.390	<0.001	1.743	[1.332–2.281]	0.583	0.139	17.523	<0.001	1.792	[1.364–2.354]	0.598	0.141	18.634	<0.001	1.843	[1.39–2.22]
Age	18–34	0.443	0.182	5.893	0.015	1.557	[1.089–2.225]	0.330	0.187	3.136	0.077	1.392	[0.965–2.006]	0.235	0.198	1.41	0.235	1.264	[0.858–1.862]
	35–54	–0.163	0.171	0.909	0.340	0.850	[0.608–1.188]	–0.120	0.174	0.476	0.490	0.887	[0.631–1.247]	–0.088	0.179	0.242	0.623	0.916	[0.645–1.300]
	≥55	Ref.						Ref.						Ref.					
Socioeconomic level	Low	0.757	0.172	19.357	<0.001	2.132	[1.522–2.987]	0.608	0.176	11.886	<0.001	1.837	[1.300–2.596]	0.564	0.180	9.824	0.002	1.758	[1.235–2.502]
	Medium	0.566	0.168	11.409	<0.001	1.761	[1.268–2.446]	0.522	0.170	9.446	0.002	1.686	[1.208–2.352]	0.469	0.174	7.277	0.007	1.598	[1.137–2.246]
	Higher	Ref.						Ref.						Ref.					
Family support	Yes							−0.641	0.196	10.725	<0.001	0.527	[0.359–0.773]	−0.633	0.200	10.012	0.002	0.531	[0.359–0.786]
	No							Ref.						Ref.					
Financial/work difficulties	Yes							0.461	0.143	10.349	<0.001	1.585	[1.197–2.099]	0.391	0.146	7.147	0.008	1.478	[1.11–1.969]
	No							Ref.						Ref.					
Trust in institutions	Low													0.421	0.146	8.324	0.004	1.523	[1.144–2.026]
	High													Ref.					
Risk perception severity	Low													−0.412	0.164	6.319	0.012	0.662	[0.48–0.913]
	High													Ref.					
Risk perception probability	Low													−0.474	0.146	10.482	<0.001	0.622	[0.467–0.829]
	High													Ref.					
Pandemic fatigue (continuous)														–0.03	0.012	5.614	0.018	0.971	[0.947–0.995]

***Note**:* WHO-5, 5-item World Health Organization Well-Being Index; SE, standard error; OR, odds ratio; CI, confidence interval; Ref, reference group.

In Round 2, the results of the final model, which are detailed in Table 4, indicated that women had a higher risk of suffering from depression (OR = 1.644; *p* <0.001) than men, as well as people with a lower socioeconomic level, who had a much higher risk (OR = 3.552; *p* < 0.001) compared to those with a higher socioeconomic level. Having support from family or friends resulted in a lower risk (OR = 0.582; *p* = 0.021) compared to not having it. Low trust in institutions indicated a higher risk (OR = 1.329; *p* = 0.046) than higher trust. Having a lower perception of the risk of COVID-19 infection resulted in a lower risk of depression (OR = 0.687; *p* = 0.010) than having a higher perception of risk. Those with a higher degree of pandemic fatigue had a higher probability of suspected depression diagnosis (OR = 1.035; *p* = 0.011), although the odds ratio results are very close to 1 so this cannot be stated categorically.

**Table 4.** **Multivariate logistic regression of the risk of depression in Round 2**.

Risk of depression according to the WHO-5 in Round 2																									
		Model 1						Model 2						Model 3						Model 4					
		B	SE	Wald	*p* value	OR	95% CI	B	SE	Wald	*p* value	OR	95% CI	B	SE	Wald	*p* value	OR	95% CI	B	SE	Wald	*p* value	OR	95% CI
Sex	Male	Ref.						Ref.						Ref.											
	Female	0.535	0.137	15.361	<0.001	1.708	[1.307–2.231]	0.516	0.138	14.072	<0.001	1.676	[1.280–2.195]	0.493	0.139	12.517	<0.001	1.637	[1.246–2.150]	0.497	0.139	12.805	<0.001	1.644	[1.252–2.159]
Age	18–34	0.578	0.167	11.964	<0.001	1.783	[1.285–2.474]	0.56	0.169	11.046	<0.001	1.751	[1.259–2.437]	0.483	0.177	7.413	0.006	1.621	[1.145–2.294]	0.429	0.174	6.047	0.014	1.535	[1.091–2.161]
	35–54	0.287	0.164	3.044	0.081	1.332	[0.965–1.839]	0.330	0.166	3.950	0.047	1.392	[1.005–1.927]	0.272	0.169	2.574	0.109	1.312	[0.942–1.829]	0.171	0.172	0.988	0.32	1.187	[0.847–1.663]
	≥55	Ref.						Ref.						Ref.											
Socioeconomic level	Low	1.419	0.201	49.913	<0.001	4.135	[2.789–6.130]	1.242	0.209	35.28	<0.001	3.463	[2.298–5.217]	1.245	0.208	35.726	0.000	3.473	[2.309–5.224]	1.268	0.208	37.204	<0.001	3.552	[2.364–5.339]
	Medium	0.544	0.17	10.224	<0.001	1.723	[1.234–2.405]	0.422	0.175	5.794	0.016	1.526	[1.082–2.152]	0.439	0.176	6.247	0.012	1.551	[1.099–2.189]	0.833	0.171	23.693	<0.001	2.301	[1.645–3.219]
	Higher	Ref.						Ref.						Ref.						Ref					
Family support	Yes							–0.666	0.231	8.332	0.004	0.514	[0.327–0.808]	–0.515	0.236	4.777	0.029	0.597	[0.376–0.948]	–0.541	0.235	5.314	0.021	0.582	[0.368–0.922]
	No							Ref.						Ref.						Ref.					
Financial/work difficulties	Yes							0.237	0.142	2.771	0.096	1.267	[0.959–1.675]												
	No							Ref.																	
Trust in institutions	Low													0.272	0.143	3.63	0.057	1.313	[0.992–1.736]	0.284	0.142	3.982	0.046	1.329	[1.005–1.757]
	High													Ref.						Ref.					
Risk perception severity	Low													–0.267	0.149	3.219	0.073	0.765	[0.572–1.025]						
	High													Ref.											
Risk perception probability	Low													–0.332	0.148	5.062	0.024	0.717	[0.537–0.958]	–0.376	0.145	6.675	0.01	0.687	[0.516–0.913]
	High													Ref.						Ref.					
Pandemic fatigue (continuous)														–0.037	0.014	7.387	0.007	1.037	[1.01–1.065]	–0.034	0.013	6.521	0.011	1.035	[1.0008–1.062]

***Note***: WHO-5, 5-item World Health Organization Well-Being Index; SE, standard error; OR, odds ratio; CI, confidence interval; Ref, reference group.

## Discussion

This study is the first to analyze subjective well-being and the influence of different sociodemographic and behavioral factors on the mental health of the population in the Region of Murcia.

In Round 1, the mean WHO-5 score was slightly higher than in Round 2, indicating that the subjective well-being of the population declined as the pandemic progressed. Using the screening tool for suspected depression, the results revealed an increase in the risk of suffering from depression (35.2% in Round 1 and 39.1% in Round 2) as the pandemic advanced. The literature highlights the heterogeneity in the prevalence of mental health impacts on the population during the COVID-19 pandemic across different regions and countries [21, 22, 23, 24, 25]. In the early stages of the pandemic, several authors reported lower prevalence of psychological distress in Spain compared to those found in this study [26], while other studies revealed findings similar to ours [27, 28].

This study has identified the most vulnerable populations for depression, which were found to be primarily associated with being female, having a low socioeconomic status, having little or no family or social support, low trust in institutions, and a high perception of the risk of COVID-19 infection, in both rounds studied.

The triple dimension of the pandemic—health, social, and economic—calls for an understanding of the gender-specific impact it produces, as it affects women and men differently due to its inherent characteristics [29]. A comprehensive systematic review and meta-analysis conducted by Santabárbara *et al*. (2021) [30] show higher levels of anxiety and poorer mental well-being in women compared to men during the pandemic. In a cohort study from 11 UK longitudinal studies, they identified unequal mental health impairment across the population, with women being more affected than men [5]. The results of our study are consistent with these findings, as women had nearly twice the risk of depression diagnosis compared to men in both rounds. These results may be influenced by the fact that women continue to perform the majority of household and caregiving tasks [31], both paid and unpaid, thereby assuming a greater mental burden as a result [29]. Furthermore, the risk of intimate partner violence against women increased during the pandemic due to the lockdown situation [32]. Ignoring the gender perspective can result in health interventions that are less effective for women at best and harmful to their well-being at worst, as shown by previous disease outbreaks like Ebola and Zika [33].

Another particularly vulnerable population is people with low financial and social resources. The results of our study show that during Round 1, the population with a lower socioeconomic level had almost twice the likelihood of suspected depression compared to those with a higher socioeconomic level, with the risk increasing to four times as the pandemic progressed (Round 2). Various studies have investigated the risk factors associated with developing depressive symptoms, anxiety, or mental health problems during the COVID-19 pandemic and have found significant associations with people of lower financial income or lower social status [34, 35, 36].

The social and political context in which the surveys were developed must be considered in terms of age. During the state of alarm, in-person educational activities were suspended in all educational institutions and across all levels in the Region of Murcia for a limited time [37], but at that time students did not experience strict lockdown and were able to maintain social contact with their peers. This may be one of the reasons why during Round 1, the youngest group did not have as high a risk of suffering from depression as in Round 2, when other social limitations and accumulated pandemic fatigue could have worsened the mental health of this group. Middle-aged adults were the first to have a higher risk of suffering from depression, likely due to the work and financial stress they were under, combined with the burden of caring for both children and parents [5, 31, 38, 39].

Having family support available if needed during potential lockdowns is associated with a lower probability of developing psychopathology in our study population. Loneliness and lack of social support have also been identified as risk factors for poorer mental health during the pandemic [40]. Our study data indicate that a high perception of risk was associated with a greater likelihood of experiencing depression, as has also been demonstrated in other studies on mental health and risk factors during the COVID-19 [41]. We have found that individuals with low trust in institutions are at higher risk of suffering from depression, as suggested by a previous study [42].

One of the most novel findings of our study was identifying the relationship between the degree of pandemic fatigue and the well-being and mental health status of our population. Although our study showed a weak association, it reflected that higher levels of pandemic fatigue were more likely to lead to depressive symptoms. Multiple studies associate pandemic fatigue with a lack of adherence to COVID-19 protective measures and the motivation to maintain them over time [43, 44].

There are several limitations to consider in this study. Firstly, data were collected through an online survey, which required participants to have internet access. Despite this, the sample was representative of the general population of Murcia in terms of age and gender. Secondly, this is a cross-sectional study, and due to the changing nature of the COVID-19 pandemic, the results refer to the specific time period in which the study was conducted. The strengths of the study include its large sample size and the use of validated scales.

## Conclusions

In this study, the risk of depression was found to be unequally distributed across the population, with certain sociodemographic characteristics and perceptions heightening that risk. The groups that are considered to be more susceptible to a risk of depression are women, people with a low socioeconomic level, those lacking support from family or friends for assistance during the pandemic, and people with a low level of trust in institutions and a high perception of the risk of COVID-19 infection.

Identifying vulnerable populations facing mental health issues can help the authorities and institutions that are responsible for managing public health crises to develop and implement inclusive strategies and interventions tailored to the population’s needs.

## Availability of Data and Materials

Data to support the findings of this study are available on reasonable request from the corresponding author.

## References

[1] Ramírez-Ortiz J, Castro-Quintero D, Lerma-Córdoba C, Yela-Ceballos F, Escobar-Córdoba F (2020). Mental health consequences of the COVID-19 pandemic associated with social isolation. *Colombian Journal of Anestesiology*.

[2] Zhang SX, Chen RZ, Xu W, Yin A, Dong RK, Chen BZ (2022). A Systematic Review and Meta-Analysis of Symptoms of Anxiety, Depression and Insomnia in Spain in the COVID-19 Crisis. *International Journal of Environmental Research and Public Health*.

[3] Luo M, Guo L, Yu M, Jiang W, Wang H (2020). The psychological and mental impact of coronavirus disease 2019 (COVID-19) on medical staff and general public - A systematic review and meta-analysis. *Psychiatry Research*.

[4] Oliver N, Barber X, Roomp K, Roomp K (2020). Assessing the Impact of the COVID-19 Pandemic in Spain: Large-Scale, Online, Self-Reported Population Survey. *Journal of Medical Internet Research*.

[5] Patel K, Robertson E, Kwong AS, Griffith GJ, Willan K, Green MJ (2022). Psychological distress before and during the COVID-19 pandemic among adults in the United Kingdom based on coordinated analyses of 11 longitudinal studies. *JAMA Network Open*.

[6] World Health Organization: Geneva (2020). The impact of COVID-19 on mental, neurological and substance use services: results of a rapid assessment. https://apps.who.int/iris/bitstream/handle/10665/335838/9789240012455-eng.pdf.

[7] Mukhtar S (2020). Psychological health during the coronavirus disease 2019 pandemic outbreak. *The International Journal of Social Psychiatry*.

[8] Regional Office for Europe (2020). Pandemic fatigue: reinvigorating the public to prevent COVID-19: policy considerations for Member States in the WHO European Region. World Health Organization. Regional Office for Europe. https://apps.who.int/iris/handle/10665/335820.

[9] Dryhurst S, Schneider C, Kerr J, Freeman A, Recchia G, van der Bles AM (2020). Risk perceptions of COVID-19 around the world. *Journal of Risk Research*.

[10] Rodríguez-Quiroga A, Buiza C, Mon MAÁ, Quintero J (2020). Update on COVID-19 and mental health. *Medicine*.

[11] Serrano Molina AM, Gómez-Sierra FJ, Fernández Ruiz J, Jiménez-Fernández S, González Domenech P, García-Jiménez J (2023). Consequences of the COVID-19 pandemic on the mental health of medical students202. *Actas Espanolas De Psiquiatria*.

[12] de Bruin M, Suk JE, Baggio M, Blomquist SE, Falcon M, Forjaz MJ (2022). Behavioural insights and the evolving COVID-19 pandemic. *Euro Surveillance: Bulletin Europeen Sur Les Maladies Transmissibles = European Communicable Disease Bulletin*.

[13] Bavel JJV, Baicker K, Boggio PS, Capraro V, Cichocka A, Cikara M (2020). Using social and behavioural science to support COVID-19 pandemic response. *Nature Human Behaviour*.

[14] WHO European Region (2020). Survey tool and guidance: rapid, simple, flexible behavioural insights on COVID-19. https://iris.who.int/bitstream/handle/10665/333549/WHO-EURO-2020-696-40431-54222-eng.pdf?sequence=1.

[15] Regional Ministry of Health of the Region of Murcia (2022). Murciasalud, the health portal of the Region of Murcia. https://www.murciasalud.es/pagina.php?id=458869.

[16] Rodríguez-Blázquez C, Romay-Barja M, Falcón M, Ayala A, Forjaz MJ (2021). The COSMO-Spain Survey: Three First Rounds of the WHO Behavioral Insights Tool. *Frontiers in Public Health*.

[17] World Health Organization (1998). Wellbeing Measures in Primary Health Care: The DepCare Project; Consensus Meeting.

[18] Topp CW, Østergaard SD, Søndergaard S, Bech P (2015). The WHO-5 Well-Being Index: a systematic review of the literature. *Psychotherapy and Psychosomatics*.

[19] Tsai FY, Schillok H, Coenen M, Merkel C, Jung-Sievers C, Cosmo Study Group (2022). The Well-Being of the German Adult Population Measured with the WHO-5 over Different Phases of the COVID-19 Pandemic: An Analysis within the COVID-19 Snapshot Monitoring Study (COSMO). *International Journal of Environmental Research and Public Health*.

[20] Rodriguez-Blazquez C, Romay-Barja M, Falcon M, Ayala A, Forjaz MJ (2022). Psychometric Properties of the COVID-19 Pandemic Fatigue Scale: Cross-sectional Online Survey Study. *JMIR Public Health and Surveillance*.

[21] Zhang SX, Miller SO, Xu W, Yin A, Chen BZ, Delios A (2022). Meta-analytic evidence of depression and anxiety in Eastern Europe during the COVID-19 pandemic. *European Journal of Psychotraumatology*.

[22] Salari N, Hosseinian-Far A, Jalali R, Vaisi-Raygani A, Rasoulpoor S, Mohammadi M (2020). Prevalence of stress, anxiety, depression among the general population during the COVID-19 pandemic: a systematic review and meta-analysis. *Globalization and Health*.

[23] Pérez-Cano HJ, Moreno-Murguía MB, Morales-López O, Crow-Buchanan O, English JA, Lozano-Alcázar J (2020). Anxiety, depression, and stress in response to the coronavirus disease-19 pandemic. *Cirugia Y Cirujanos*.

[24] Bareeqa SB, Ahmed SI, Samar SS, Yasin W, Zehra S, Monese GM (2021). Prevalence of depression, anxiety and stress in China during COVID-19 pandemic: A systematic review with meta-analysis. *The International Journal of Psychiatry in Medicine*.

[25] Krishnamoorthy Y, Nagarajan R, Saya GK, Menon V (2020). Prevalence of psychological morbidities among general population, healthcare workers and COVID-19 patients amidst the COVID-19 pandemic: A systematic review and meta-analysis. *Psychiatry Research*.

[26] González-Sanguino C, Ausín B, Castellanos MÁ, Saiz J, López-Gómez A, Ugidos C (2020). Mental health consequences during the initial stage of the 2020 Coronavirus pandemic (COVID-19) in Spain. *Brain, Behavior, and Immunity*.

[27] Planchuelo-Gómez Á, Odriozola-González P, Irurtia MJ, de Luis-García R (2020). Longitudinal evaluation of the psychological impact of the COVID-19 crisis in Spain. *Journal of Affective Disorders*.

[28] Rodríguez-Rey R, Garrido-Hernansaiz H, Collado S (2020). Psychological Impact and Associated Factors During the Initial Stage of the Coronavirus (COVID-19) Pandemic Among the General Population in Spain. *Frontiers in Psychology*.

[29] (2020). The gender approach, key in COVID-19 response. Catalogue of publications of the General State Administration. https://www.inmujeres.gob.es/diseno/novedades/PlantillaCovid-19/EN_IMPACTO_DE_GENERO_DEL_COVID-19_03_EN.pdf.

[30] Santabárbara J, Lasheras I, Lipnicki DM, Bueno-Notivol J, Pérez-Moreno M, LópezAntón R (2021). Prevalence of anxiety in the COVID-19 pandemic: An updated meta-analysis of community-based studies. *Progress in Neuro-Psychopharmacology & Biological Psychiatry*.

[31] Li R, Karukivi M, Lindblom J, Korja R, Karlsson L, Karlsson H (2024). Trajectories of COVID-19 pandemic-related depressive symptoms and potential predictors: the FinnBrain Birth Cohort Study. *Social Psychiatry and Psychiatric Epidemiology*.

[32] Mazza M, Marano G, Lai C, Janiri L, Sani G (2020). Danger in danger: Interpersonal violence during COVID-19 quarantine. *Psychiatry Research*.

[33] Davies SE, Bennett B (2016). A gendered human rights analysis of Ebola and Zika: locating gender in global health emergencies. *International Affairs*.

[34] Mazza C, Ricci E, Biondi S, Colasanti M, Ferracuti S, Napoli C (2020). A Nationwide Survey of Psychological Distress among Italian People during the COVID-19 Pandemic: Immediate Psychological Responses and Associated Factors. *International Journal of Environmental Research and Public Health*.

[35] Wang Y, Di Y, Ye J, Wei W (2021). Study on the public psychological states and its related factors during the outbreak of coronavirus disease 2019 (COVID-19) in some regions of China. *Psychology, Health & Medicine*.

[36] Qi Y, Lepe A, Almansa J, Ots P, de Kroon MLA, Lifelines Corona Research Initiative (2022). Increases in symptoms of depression and anxiety in adults during the initial phases of the COVID-19 pandemic are limited to those with less resources: Results from the Lifelines Cohort Study. *Journal of Psychiatric Research*.

[37] (2020). Presidential Decree No 11/2020 of 22 December updating the restrictive measures adopted under Royal Decree 926/2020 of 25 October declaring a state of alarm to contain the spread of infections caused by SARS-COV-2. BORM nº 296. https://www.borm.es/#/home/anuncio/23-12-2020/7470.

[38] Jorm AF, Windsor TD, Dear KBG, Anstey KJ, Christensen H, Rodgers B (2005). Age group differences in psychological distress: the role of psychosocial risk factors that vary with age. *Psychological Medicine*.

[39] Horesh D, Kapel Lev-Ari R, Hasson-Ohayon I (2020). Risk factors for psychological distress during the COVID-19 pandemic in Israel: Loneliness, age, gender, and health status play an important role. *British Journal of Health Psychology*.

[40] Palgi Y, Shrira A, Ring L, Bodner E, Avidor S, Bergman Y (2020). The loneliness pandemic: Loneliness and other concomitants of depression, anxiety and their comorbidity during the COVID-19 outbreak. *Journal of Affective Disorders*.

[41] Olagoke AA, Olagoke OO, Hughes AM (2020). Exposure to coronavirus news on mainstream media: The role of risk perceptions and depression. *British Journal of Health Psychology*.

[42] Hensel L, Witte M, Caria AS, Fetzer T, Fiorin S, Götz FM (2022). Global Behaviors, Perceptions, and the Emergence of Social Norms at the Onset of the COVID-19 Pandemic. *Journal of Economic Behavior & Organization*.

[43] MacIntyre CR, Nguyen PY, Chughtai AA, Trent M, Gerber B, Steinhofel K (2021). Mask use, risk-mitigation behaviours and pandemic fatigue during the COVID-19 pandemic in five cities in Australia, the UK and USA: A cross-sectional survey. *International Journal of Infectious Diseases: IJID: Official Publication of the International Society for Infectious Diseases*.

[44] Petherick A, Goldszmidt R, Andrade EB, Furst R, Hale T, Pott A (2021). A worldwide assessment of changes in adherence to COVID-19 protective behaviours and hypothesized pandemic fatigue. *Nature Human Behaviour*.

